# Experimental Study of Rheological Properties and Oil Displacement Efficiency in Oilfields for a Synthetic Hydrophobically Modified Polymer

**DOI:** 10.1038/s41598-017-09057-9

**Published:** 2017-08-18

**Authors:** Pengcheng Liu, Zhenbao Mu, Chao Wang, Yanling Wang

**Affiliations:** 1 School of Energy Resources, China University of Geosciences, 29 Xueyuan Road, Beijing, 100083 China; 2 Research Institute of Petroleum Exploration and Development, SINOPEC, 31 Xueyuan Road, Beijing, 100083 China; 3 School of Petroleum Engineering, Petroleum University of China, 66 Changjiang West Road, Qingdao, 266555 China

## Abstract

In a previous study, we developed a synthetic hydrophobically modified hydroxyethyl cellulose (HEC) using bromododecane (BD), which we denote as BD-HMHEC. In this work, we continually investigate the rheological properties and its oil displacement efficiency in PuTao well area in Daqing oilfields, China. Results show that BD-HMHEC solution has good viscosification, thermal-resistance, salt-tolerance, shear resistance, and acid/alkali resistance. The storage modulus (*G’*) and the loose modulus (*G*”) of the BD-HMHEC solutions increase significantly with increasing BD-HMHEC concentration, and the solution becomes viscoelastic at a sufficiently high BD-HMHEC concentration. The core flooding results showed BD-HMHEC flooding improves oil recovery by 7–14% in comparison with HEC flooding at concentrations of 4,000 mg/L under equivalent conditions. Moreover, BD-HMHEC flooding improves oil recovery by 7–8% after conducting water and hydrolyzed polyacrylamide (HPAM) flooding. The oil displacement mechanism of BD-HMHEC solutions is discussed based on a visual evaluation. The results indicate that BD-HMHEC flooding is a feasible means for improving oil recovery after water/HPAM flooding.

## Introduction

Hydrophobically modified polymers (HM-polymers) represent a class of water-soluble polymers, where a small number of hydrophobic groups are introduced to the macromolecular chain of conventional water-soluble polymers^1, 2^. When an HM-polymer is dissolved in water, supramolecular aggregates and a reversible network structure are formed owing to association among the hydrophobic groups; thus, the solution viscosity increases significantly^3–5^. As such, HM-polymers are similar to the conventional polymers (e.g., hydrolyzed polyacrylamide (HPAM)) used extensively in the field of oil recovery. However, HM-polymers usually exhibit unique rheological properties, and also demonstrate good thermal-resistance, salt-tolerance, shear resistance, and acid/alkali resistance^6^. All types of HM-polymers are suitable substitutes for HPAM as oil displacement and profile modification agents for high temperature and high salinity reservoirs^7, 8^. HM-polymers have been reviewed in detail with particular emphasis on their application during enhanced oil recovery (EOR) processes^9–12^.

The rheology and oil displacement characteristics of hydrophobically modified polyacrylamide polymer (HMPAM) have been widely studied^13–16^. Hydrophobically modified hydroxyethyl cellulose (HEC), denoted as HMHEC, is another significant synthetic compound that has been widely used in various applications^17–23^. HMHEC has been claimed to have potential in EOR processes^11^.

Compared with HPAM, HEC provides a wide range of raw material sources that are non-toxic and have better properties, such as thickening and biocompatibility. In addition, the unique structure and properties of HEC ensure that it is easily modified chemically, making it useful for manufacturing various polymers. Therefore, it is of prospective and practical significance to replace toxic and non-degradable synthetic polymers with HEC in EOR processes^24–26^.

However, to the best of authors’ knowledge, previous research regarding HMHEC has mainly focused on the laboratory evaluation of the rheological performance and unique properties of HMHEC solutions, and consideration of its use as a good oil displacement system to enhance oil recovery has been neglected^27–29^. In previous work, we developed a synthetic HMHEC by the macromolecular reaction between HEC and the long chain alkyl halides of bromododecane (BD), herein denoted as BD-HMHEC, which focused on the development of material with enhanced properties^30^. In this study, the main goal of the first section was used to further consider its rheological properties, which give scientific understanding of the structure property-relationships between the thickening apparent viscosity and rheological parameters. The main goal of the second section was used to investigate its oil displacement efficiency, which describes the different effects on flooding processes between the rheological performance and EOR. To understand the different effects of HPAM, HEC, and BD-HMHEC on flooding processes, the oil displacement performances of these water-soluble polymers were evaluated by core flooding based on field sampling, the actual formation pressure and temperature from the PuTao well area in Daqing oilfields (China). Then, the oil displacement mechanism of BD-HMHEC solutions was discussed according to a visual evaluation. The results of core flooding indicate that BD-HMHEC has much better oil displacement properties than HPAM and HEC, and has great potential in EOR processes.

## Experimental

### Preparation of polymer solutions

A given volume of distilled water was placed in a beaker, and a given amount of polymer powder was slowly added while rapidly stirring with a stirring device. The stirring speed was reduced appropriately until the polymer was completely dissolved, and the solution was then stored for 24 h at room temperature. Polymer solution was prepared for application.

### Method for determination of apparent viscosity of polymer solutions

The apparent viscosity (*u*_a_) of the polymer solution was directly read using a DV-II + Pro rotor viscometer (Brookfield, US), whose accuracy is ±1.0% and range repeatability is ±0.2%. Unless otherwise stated, testing was conducted with a shear rate of 6 s^−1^ at a temperature of 25 ± 0.1 °C.

### Method for determination of *G’* and *G”*

The viscoelastic properties of a polymer solution are represented by a combination of viscous and elastic characteristics, and are observable as a response to an applied force. Dynamic viscoelasticity is observable as a response to an oscillating strain of a given frequency imposed on the polymer solution in a non-destructive state, and the viscosity and elasticity are characterized by the sizes of *G*′ and *G*″ in the solution. The values of *G*′ and *G*″ of polymer solutions were measured using a Physica MCR 301 coaxial rotary rheometer (Anton Paar) with concentrations of 3000 mg/L, 4000 mg/L, 6000 mg/L, and 8000 mg/L. The oscillation frequency range was from 0.01–100 Hz at 25 °C.

### Oil displacement experimental equipment and procedure

**Figure 1:**
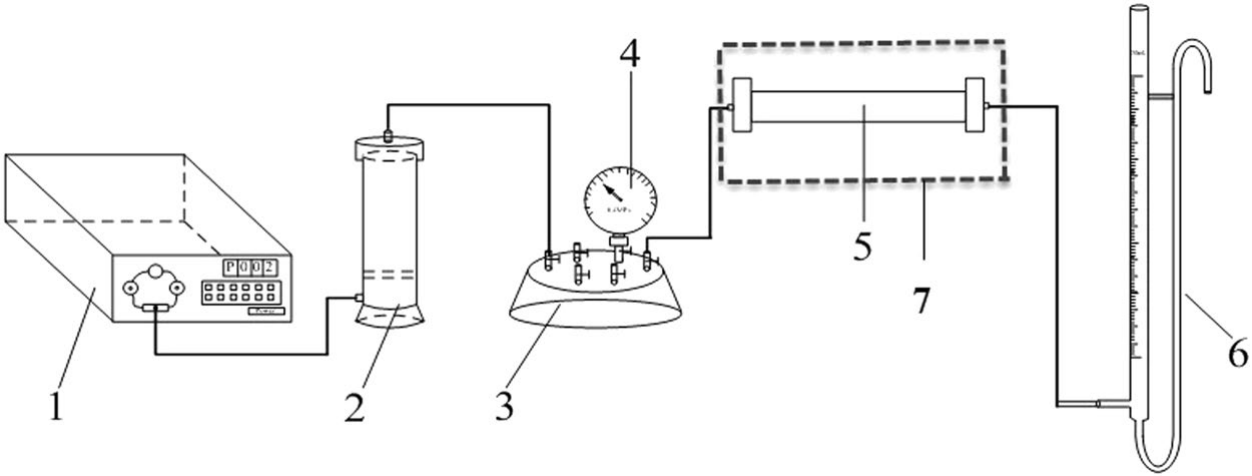
Oil displacement experimental apparatus. 1 Constant-flux pump. 2 Accumulator; 3 Six-way valve. 4 Pressure gauge; 5 Core holder. 6 Oil-water separation pipe. 7 Constant-temperature oven.

Figure 1 shows a schematic of the experimental set-up for oil displacement experiments. The test procedure was as follows.

1. The core holder (5, in Fig. 1) was packed with actual cores from the PuTao well area whose physical features and geometrical dimensions are listed in Table 1, Table 2, and Table 3 and its weight was measured.

**Table 1:** The results of HPAM and BD-HMHEC flooding after water flooding (injection rate: 0.5 mL/min).

Core No.	Polymer	Length (cm)	Section area (cm^2^)	Permeability (µm^2^)	Porosity (%)	Injected slug (PV)	Incremental oil recovery (%)		
							I phase	II phase	III phase
							Water flooding	HPAM flooding	BD-HMHEC flooding
1-a#	HPAM/BD-HMHEC (4000 mg/L)	30	4.676	1.812	33.64	0.3	35.7	3.1	10.3
1-b#		30	4.676	1.817	34.05	0.4	36.1	4.5	15.4
1-c#		30	4.676	1.809	33.26	0.5	35.9	5.3	18.4
1-d#		30	4.676	1.820	33.94	0.6	35.6	6.2	20.1
1-e#		30	4.676	1.819	33.56	0.7	36.2	6.3	21.5

**Table 2:** The results of HPAM and HEC flooding after water flooding (injection rate: 0.5 mL/min).

Core No.	Polymer	Length (cm)	Section area (cm^2^)	Permeability (µm^2^)	Porosity (%)	Injected slug (PV)	Incremental oil recovery (%)		
							I phase	II phase	III phase
							Water flooding	HPAM flooding	HEC flooding
2-a#	HPAM/HEC (4000 mg/L)	30	4.676	1.821	34.02	0.3	36.4	3.0	3.6
2-b#		30	4.676	1.812	34.16	0.4	35.9	4.3	4.9
2-c#		30	4.676	1.819	33.96	0.5	37.5	5.5	6.0
2-d#		30	4.676	1.825	35.09	0.6	37.1	6.4	7.3
2-e#		30	4.676	1.809	33.82	0.7	36.2	6.2	7.9

**Table 3:** The results of continuing HPAM and switching to BD-HMHEC flooding after water and HPAM flooding (injection rate: 0.5 mL/min).

Core No.	Polymer concentration (mg/L)		Permeability (µm^2^)	HPAM Injected slug (PV)	Second HPAM/ BD-HMHEC Injected slug (PV)	Incremental oil recovery (%)			
						I phase	II phase	III phase	
	HPAM	BD-HMHEC				Water flooding	HPAM flooding	Switching to BD-HMHEC flooding	Continuing HPAM flooding
3-a#	1250	4000	1.905	0.5	0.5	36.9	16.6	—	2.4
3-b#	1250	4000	1.910	0.5	0.5	37.0	16.8	7.9	—
4-a#	1250	4000	5.139	0.5	0.5	53.5	12.9	—	2.1
4-b#	1250	4000	5.180	0.5	0.5	53.8	13.0	7.3	—
2. The core was saturated with formation water, aged for about 4 h, and then formation water was injected at a rate of 0.5 mL/min into the core. The pressures at both ends of the core were measured and the core’s permeability to brine was determined employing Darcy’s law. The weight of the saturated core and its porosity were measured employing the saturated weighing method (in Table 1, Table 2, and Table 3).
3. Crude oil was injected into the core until water was no longer produced, and the irreducible water saturation was calculated. The core was aged at 60 °C for 24 h and loaded into the core holder and placed into constant temperature oven. The oil displacement experiments were conducted until the oven temperature reached 60 °C (actual formation temperature of the PuTao well area).
4. Water was then injected until the water cut reached 98%, and the water flooding recovery was calculated.
5. A polymer solution was injected until the water cut exceeded 98%, and the oil recovery of polymer solution flooding was calculated.

### Visual evaluation experimental equipment and procedure

**Figure 2:**
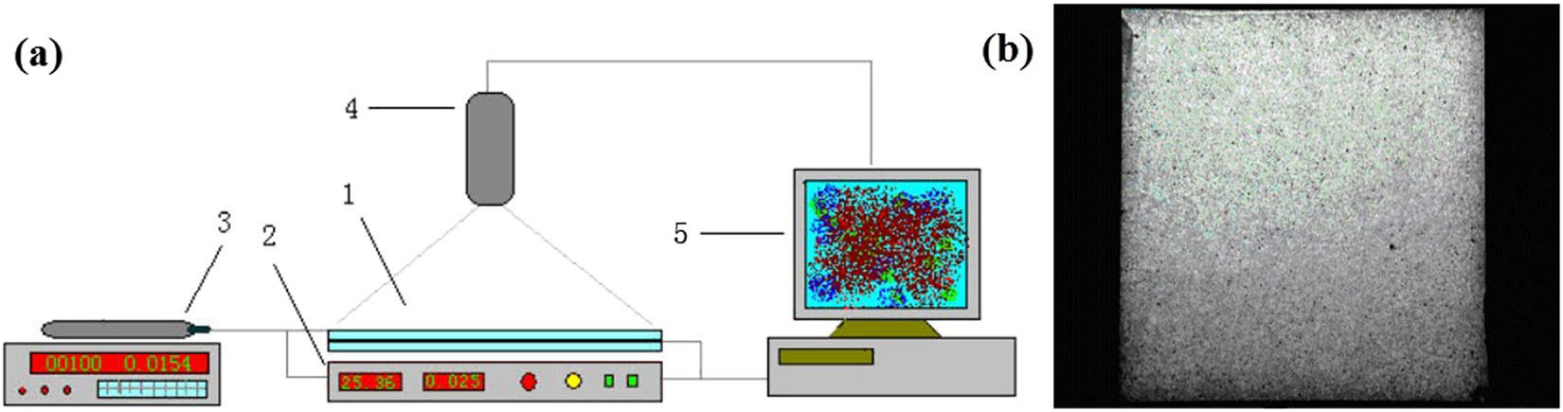
Device and physical model for visual evaluation of oil displacement. (**a**) Device for visually evaluating oil displacement. 1 Transparent visualization model. 2 Thermostatic physical model tank bottom. 3 Micro pump filled with fluid. 4 Camera for continuous, real-time recording of various substances. 5 Computer analyzed image data. (**b**) Physical model of visualization.

Figure 2 shows the visual valuation experimental equipment was employed to evaluate oil displacement efficiency. The equipment is comprised of a transparent and flat plate models, thermostatic physical model tank bottom, micro pump filled with fluid, camera for continuous, real-time recording of various substances, computer analyzed image data system, and other components (in Fig. 2a). The right amount of epoxy evenly was sparingly applied evenly over both surfaces of the parallel glass in the transparent and flat plate models. The quartz sand or natural core powder was spread on the rubber surface to ensure uniformity and smoothness (in Fig. 2b). The experimental procedure is described as follows.

1. The transparent model was saturated with formation water and then crude oil was injected until no water was produced. The model was installed in the experimental equipment.
2. Water was injected at a rate of 0.15 mL/min using a micro pump and the digital camera device and computer analyzed image data system started to record.
3. When the water cut exceeded 98% from the outlet model, water flooding was stopped together with the micro pump, camera device and computer system.
4. BD-HMHEC solution was switched to inject at the same flow rate using the micro pump and the digital camera device and computer analyzed image data system started to record again.
5. When the water cut exceeded 98%, BD-HMHEC was finished together with the micro pump, camera device and computer system.
6. The captured images and measured data were saved after all the evaluation experiments.

## Results and Discussion

### Viscosification of the BD-HMHEC solution

#### Effect of polymer concentration on *u*_a_

The apparent viscosity (*u*_a_) of BD-HMHEC and HEC solutions were read with concentrations of 500 mg/L, 1000 mg/L, 2000 mg/L, 4000 mg/L, 6000 mg/L, 8000 mg/L and 10000 mg/L (temperature: 25 °C; shear rate: 6 s^−1^).

**Figure 3:**
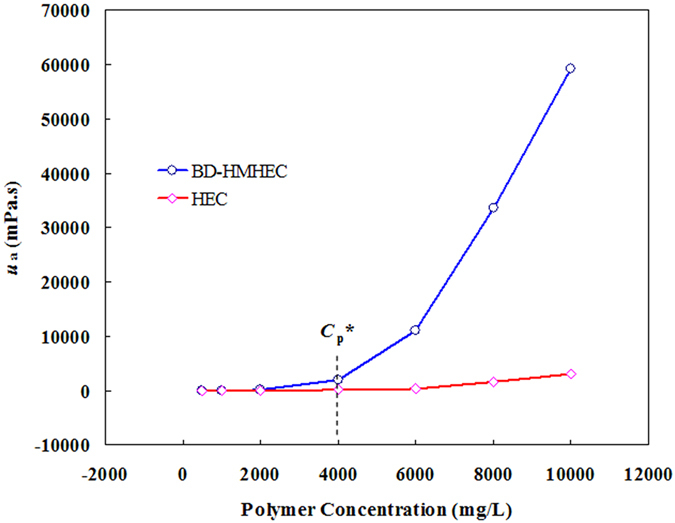
Effect of polymer concentration on *u*_a_ of BD-HMHEC and HEC solutions.

The trends with which the *u*_a_ of BD-HMHEC and HEC solutions vary with respect to their concentrations shown in Fig. 3 are similar to that of HMPAM solutions^15, 31, 32^.

From Fig. 3, when the concentration of the BD-HMHEC solution was below the critical association concentration (*C*_p_^*^; 4000 mg/L), the *u*_a_ value was not significantly different from that of the HEC solution with concentrations of 500 mg/L, 1000 mg/L and 2000 mg/L. At concentrations below *C*_*p*_, polymer molecules in the solution are mainly intramolecular-associated, and the molecular chains tend to shrink, resulting in a smaller *u*_a_. When the concentration reached *C*_p_^*^, the *u*_a_ of the BD-HMHEC solution rose sharply to 2,040 mPa·s, while HEC remained at only 93 mPa·s. With increasing concentration above *C*_p_^*^, the *u*_a_ of the BD-HMHEC solution increased much more rapidly than that of the HEC solution because the BD-HMHEC solution has a supramolecular agglomerate structure that enlarges the hydrodynamic volume at and above *C*_p_^*^, resulting in a notable increase in *u*_a_^33^. BD-HMHEC solutions can obtain higher *u*_a_ than HEC solutions at equivalent concentrations, and BD-HMHEC can been widely applied in EOR processes.

#### Effect of temperature on *u*_a_

**Figure 4:**
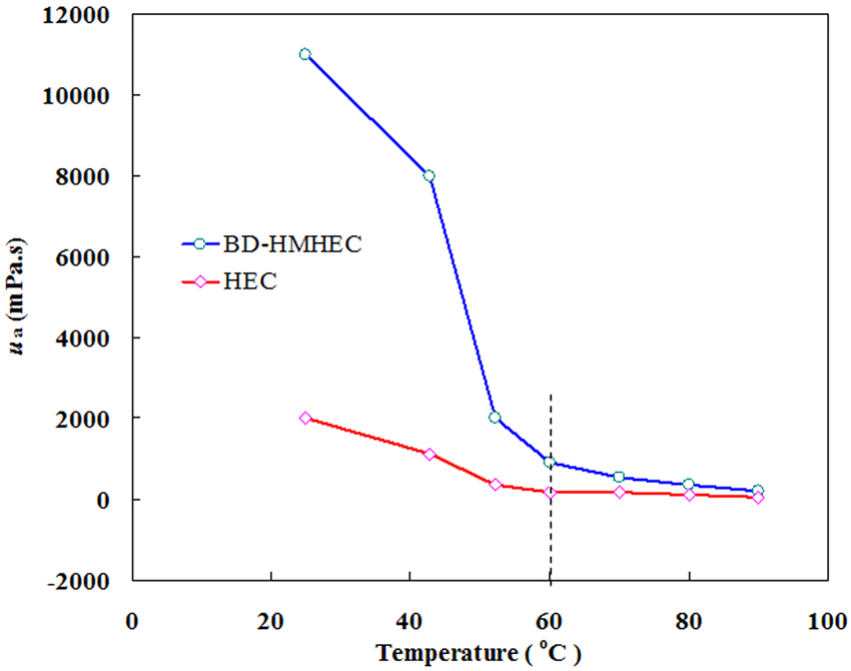
Effect of temperature on *u*_a_ of BD-HMHEC and HEC solutions.

Figure 4 shows the effect of temperature on the *u*_a_ values of BD-HMHEC and HEC solutions (concentration: 6000 mg/L; shear rate: 6 s^−1^).

From Fig. 4, the *u*_a_ values of the BD-HMHEC and HEC solutions both greatly decreased with increasing temperature, and decreased much more slowly for temperatures above 60 °C, where both attained a nearly stable value. When the temperature reached 90 °C, the nearly stable *u*_a_ value of the BD-HMHEC solution was only a small greater than that of HEC solution. The greater *u*_a_ of the BD-HMHEC solution than that of HEC solution at a certain temperatures (less than 60 °C) is attributed to the intermolecular associations due to the endothermic process of entropy increase for hydrophobic association. The reason for the *u*_a_ value of the BD-HMHEC solution greatly decreasing is attributed to intensify the thermal motion of the hydrophobic group, weaken the hydration of the hydrophobic group, and reduce the hydrodynamic volume with increasing temperature^15, 34^. However, the nearly stable value of the BD-HMHEC solution was greater than that of HEC, which illustrates that BD-HMHEC provided some improvements in the thermal-resistance performance.

These results suggest that BD-HMHEC solution would be more effective than HEC solution in high temperature (60 ~ 90 °C) reservoir applications.

#### Effect of shear rate on *u*_a_

**Figure 5:**
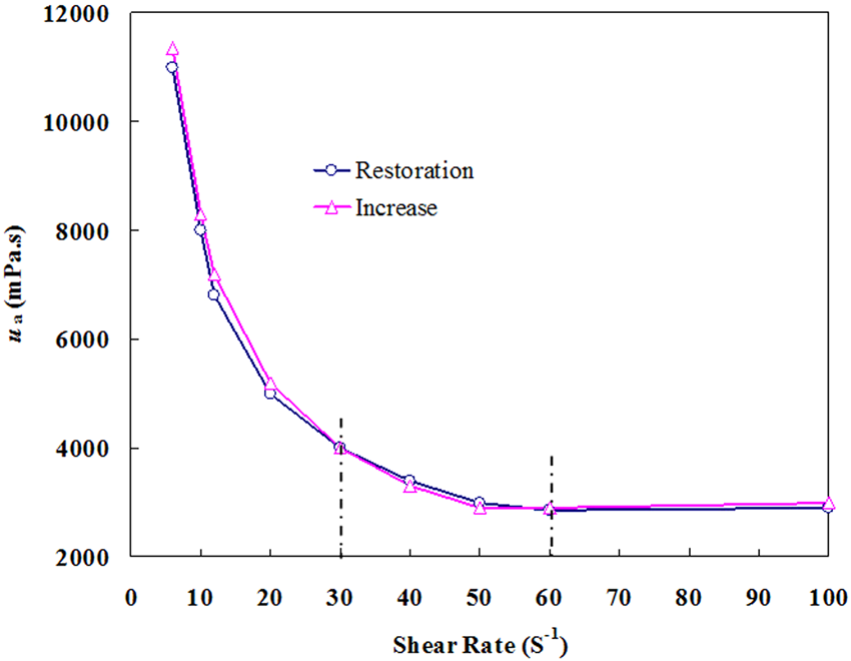
Effect of the shear rate on *u*_a_ of a BD-HMHEC solution.

Figure 5 shows the effect of the shear rate on the *u*_a_ value of a BD-HMHEC solution (concentration: 6000 mg/L; temperature: 25 °C). *u*_a_ is observed to decrease with increasing shear rate, and increase during restoration of the initial shear rate value over a range of 6–100 s^−1^. Special note, the relative error of hysteresis between increase and restoration processes was larger than the experimental error of the measurements (DV-II + Pro rotor viscometer: Accuracy is ±1.0%; Range repeatability is ±0.2%) in these experiments.

From Fig. 5, for shear rate values in the range of 30–60 s^−1^, the *u*_a_ values are somewhat larger during the shear rate restoration phase than that obtained during the initial increase. The results indicate that the BD-HMHEC molecules gradually form more comprehensive supramolecular agglomeration networks during shear rate restoration, and, consequently, the *u*_a_ is not only recovered, but is generally somewhat larger. Specifically, for shear rate values below 30 s^−1^, the *u*_a_ values are somewhat smaller during the shear rate restoration phase than that obtained during the initial increase. The *u*_a_ of the BD-HMHEC solution ultimately failed to return to its initial value mainly because, after being subjected to shearing action, the degree of network sophistication required a relaxation time to return to its original level, which represented the observed time hysteresis.

The supramolecular agglomeration networks of the BD-HMHEC solution presented a dynamic equilibrium between association and disassociation, which has some time-dependency, and it is not instantly completed. The results show that the BD-HMHEC solution exhibits good shear-resistance, in which the molecular structure is stable upon subjection to an increasing shear rate. Therefore, the application of BD-HMHEC is suitable for EOR processes in medium- and high-permeability reservoirs.

#### Effect of NaCl on *u*_*a*_

To simplify the discussion, only the effect of NaCl concentration on *u*_a_ was considered in this paper, although other more complicated factors (such as salting-in or salting-out effects) may exhibit different effects on the properties of BD-HMHEC and HEC solutions.

**Figure 6:**
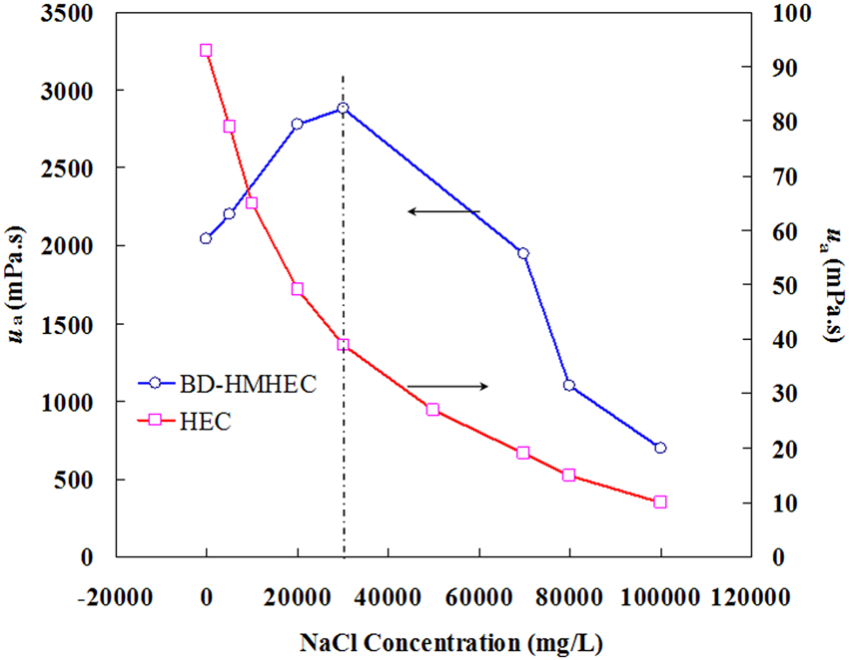
Effect of NaCl concentration on *u*_a_ of BD-HMHEC and HEC solutions.

Figure 6 shows the effect of the NaCl concentration on the *u*_a_ values of BD-HMHEC and HEC solutions (concentration: 4000 mg/L; temperature: 25 °C; shear rate: 6 s^−1^). From Fig. 6, the *u*_a_ values of the HEC solution decreased monotonically with increasing NaCl concentration. It is well known that the main drawback of HEC (or HPAM) is its sensitivity to salt, while a hydrophobically associated polymer responds differently owing to the introduction of hydrophobic groups.

As shown in Fig. 6, the *u*_a_ values of the BD-HMHEC solution increased with increasing NaCl concentration for an NaCl concentration less than 30,000 mg/L. The addition of electrolytes enhances the polarity of the solution environment, and the hydrophobic response of the non-polarized hydrophobic groups becomes substantial, which is prone to form hydrophobic micro-areas. While the hydration of the hydrophobic groups becomes thin, the system forms a more complete and wider supramolecular network structure, causing the volume fluid mechanics to further increase, and the *u*_a_ of the BD-HMHEC solution increases^35^.

For NaCl concentrations above 30,000 mg/L, higher salt concentration drives the addition of electrolytes and stronger hydrophobic association action, which can led to separation of the association. The solution displays slightly cloudy and partial polymers precipitated out of the solution, and *u*_a_ decreased rapidly with increasing NaCl concentration. However, the *u*_a_ of the BD-HMHEC solution was still 699.9 mPa·s and the *u*_a_ of the HEC solution was only 10.0 mPa·s at NaCl concentration of 100,000 mg/L. BD-HMHEC solution represents good salt-tolerance and is therefore effective for EOR under high salinity (<100000 mg/L) reservoir conditions.

#### Effect of pH on *u*_*a*_

**Figure 7:**
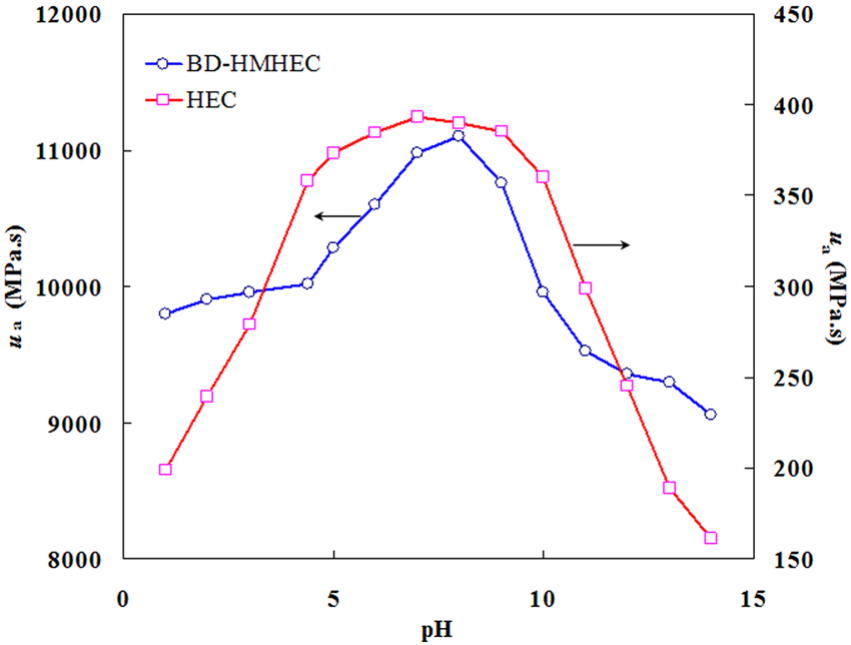
Effect of pH on *u*_a_ of BD-HMHEC and HEC solutions.

Figure 7 shows the effect of the pH value on the *u*_a_ of BD-HMHEC and HEC solutions (concentration: 6000 mg/L; temperature: 25 °C; shear rate: 6 s^−1^).

From Fig. 7, when the pH was between 4 and 10, the *u*_a_ of the HEC solution exhibited little change, and, when HEC was in an acid/alkali environment, its *u*_a_ declined sharply. However, the *u*_a_ of the BD-HMHEC solution was as high as 11,000 mPa·s in a nearly neutral environment, and remained above 9000 mPa·s in an acid/alkali environment. This indicates that the BD-HMHEC solution exhibited a degree of acid/alkali resistance.

As shown in Fig. 7, in a nearly neutral environment, the electrostatic repulsion of intermolecular polymer is minimal, and the macromolecular chains begin to extend; therefore, the *u*_a_ of a polymer attains a maximum value. In a strong acidic environment, with higher H^+^ concentration, the polymer associates the hydrogen ion to form an electrostatic repulsion of the intermolecular polymer, and the intermolecular action becomes weak, resulting in a decrease in *u*_a_. When the solution environment becomes alkaline, electrostatic repulsion of the intermolecular polymer increases, and the intermolecular action decreases with increasing OH^−^ concentration. The molecular chain of a polymer may become damaged in an alkaline environment, which leads to a rapid decrease in *u*_a_.

Whether under a nearly neutral or an acid/alkali reservoir environment, the acid/alkali resistance of BD-HMHEC can widen its application scope for EOR.

### Viscoelasticity of the BD-HMHEC solution

**Figure 8:**
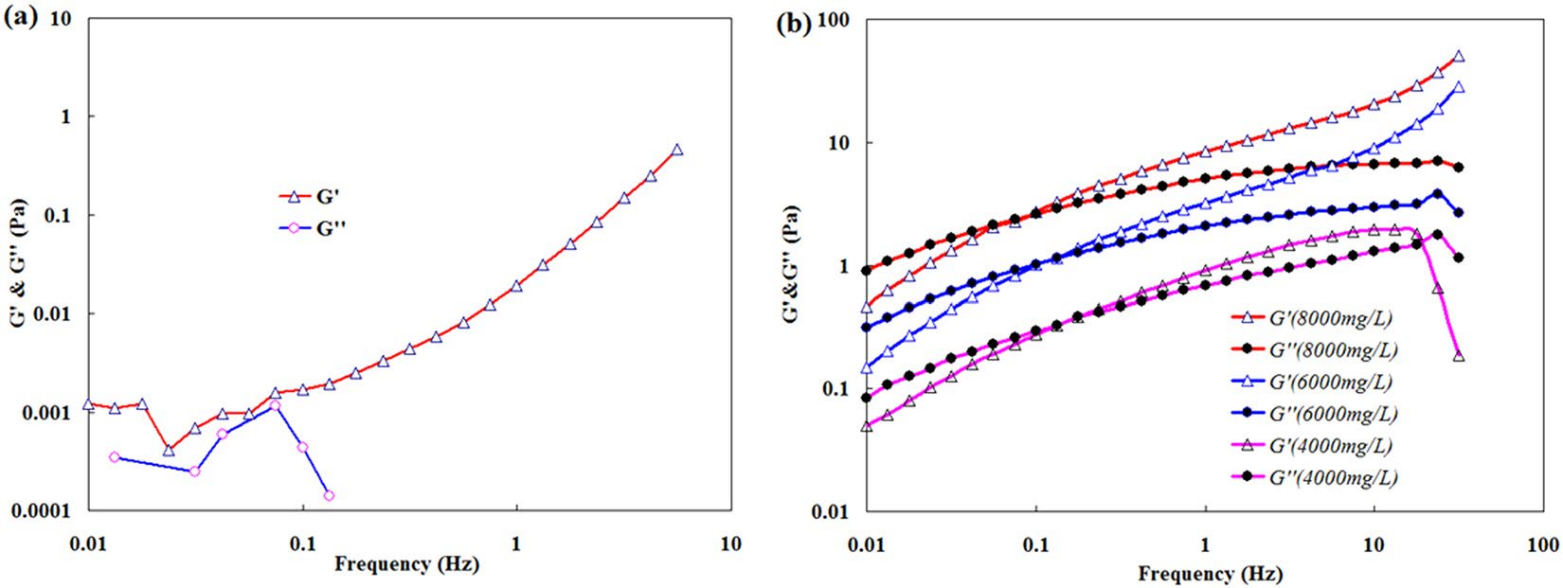
Viscoelastic curves of BD-HMHEC solutions of different concentrations. (**a**) BD-HMHEC concentration: 3000 mg/L. (**b**) BD-HMHEC concentrations: 4000 mg/L, 6000 mg/L, and 8000 mg/L.

Figure 8 shows the viscoelastic curves of BD-HMHEC solutions at different concentrations.

Figure 8(a) indicates that, when the BD-HMHEC concentration was low (3000 mg/L), the solution exhibited no viscoelasticity. One reason for this finding is that the interaction of the molecular chains is weak, and the inter-molecular chains exhibit no obvious entanglement. The other reason is that the hydrophobic association action of the solution is also weak, and the intramolecular association becomes dominant, resulting in a failure to form a spatial network aggregate structure.

Figure 8(b) indicates that *G*′ and *G*″ increased with increasing BD-HMHEC concentration (4000 mg/L, 6000 mg/L, and 8000 mg/L) at the same frequency, and that the viscoelasticity of the solution became increasingly obvious with increasing polymer concentration. Viscoelasticity was exhibited when the BD-HMHEC concentration was 4000 mg/L (i.e., at *C*_p_^*^), and *G*′ and *G*″ were observed to increase with increasing oscillation frequency. For a BD-HMHEC concentration of *C*_p_^*^, the intermolecular action replaces intramolecular association with intermolecular association, and the BD-HMHEC molecules begin to form a supramolecular network structure, resulting in a more obvious viscoelasticity.

Similar to a hydrophobically associated polymer, the strength of the entanglement action of the molecule chain increases with increasing BD-HMHEC concentration. Moreover, the viscoelasticity of the hydrophobically associated polymer becomes increasingly obvious as the hydrophobic association action of the molecule chain increases. The *G*′ and *G*″ values exhibit a crossing point for each concentration, which is denoted as the specified frequency (SF). *G*″ is greater than *G*′ when the frequency is less than the SF, while *G’* is greater than *G”* when the frequency is greater than the SF. At a relatively low frequency range, the viscous component dominates the viscoelastic properties of a BD-HMHEC solution^36^. However, when the frequency is greater than the SF, the elasticity component becomes the dominate factor.

Figure 8(b) also shows that the SF point decreases with increasing polymer concentration. Also, the corresponding elasticity of a high polymer concentration solution exceeds the corresponding viscosity at a lower frequency. At a large concentration, the molecules of BD-HMHEC solution are prone to form hydrophobic micro-areas, which serve as a basic connection to form a larger supramolecular network structure^33^.

Therefore, the drive toward network structure formation increases with increasing BD-HMHEC concentration, so that the SF decreases with increasing BD-HMHEC concentration.

### Oil displacement efficiency

In order to understand the different effects of HPAM, HEC, and BD-HMHEC solutions on core flooding processes, the similar core physical properties to their parent were prepared to avoid cleaning the core after each injection experiment.

We selected to cut several different small-size medium-permeability core samples (Core No. 1-a#, 1-b#, 1-c#, 1-d# and 1-e# for BD-HMHEC flooding; 2-a#, 2-b#, 2-c#, 2-d# and 2-e# for HEC flooding) with the section area of 4.676 cm^2^ and the length of 30.00 cm based on field samples from the PuTao well area in Daqing oilfields (China). These core samples were basically uniformly cut from the same period of the full-diameter cores (Tables 1, 2 and 3).

#### Core displacement experiment of the BD-HMHEC solution

The BD-HMHEC concentration of the solution employed in the experiment was 4,000 mg/L (injection rate: 0.5 mL/min). Core flooding tests were conducted to evaluate the effects of the HPAM and BD-HMHEC flooding on oil recovery after water flooding.

Core flooding tests were divided into three phases: I phase was conducted water flooding; II phase was conducted HPAM flooding and III phase was conducted BD-HMHEC flooding. Table 1 lists the experimental results of three phases with injected different PV (Pore Volume) slugs of HPAM (II phase) and BD-HMHEC (III phase) flooding and water flooding (I phase) in low permeability cores.

From Table 1, the results indicate the incremental oil recovery of HPAM and BD-HMHEC flooding improved that of water flooding by 3.0–6.0% and 10–20%, respectively. BD-HMHEC solution exhibits good EOR performance than HPAM solution under equivalent conditions. In addition, it was determined that the oil recovery of BD-HMHEC flooding increased significantly with increasing injected slug sizes in the range 0.3–0.5 PV. The oil recovery of BD-HMHEC flooding increased slightly for injected slug sizes greater than 0.5 PV.

#### Core displacement experiment of the HEC solution

Core flooding tests were also divided into three phases: I phase was conducted water flooding; II phase was conducted HPAM flooding and III phase was conducted HEC flooding. Table 2 lists the experimental results of three phases with different PV slugs for HPAM (II phase) and HEC (III phase) flooding at the concentration of 4,000 mg/L (injection rate: 0.5 mL/min) after water flooding (I phase) in low permeability cores.

From Table 2, the results indicate that the absolute incremental oil recovery of HPAM and HEC flooding improved that of water flooding by 3–6% and 3–8%, respectively.

Comparison of Table 1 with Table 2 indicates that the absolute incremental oil recovery of BD-HMHEC flooding was about 7–14% higher than that of HEC flooding under equivalent conditions.

#### EOR of continuing HPAM and switching to BD-HMHEC after water/HPAM flooding

To avoid cleaning the core after each injection experiment, we selected to cut two small-size medium-permeability core samples (Core No. 3-a#, 3-b#) and two small-size high-permeability core samples (Core No. 4-a#, 4-b#) with the section area of 4.676 cm^2^ and the length of 30.00 cm (Table 3).

To further investigate the oil displacement performance of the BD-HMHEC flooding, core flooding tests were also divided into three phases: I phase was conducted water flooding; II phase was conducted HPAM flooding and III phase was conducted “Switching to BD-HMHEC flooding” or “Continuing HPAM flooding”.

Table 3 lists the results of the “Continuing HPAM flooding” and “Switching to BD-HMHEC flooding” after conducting water and conventional HPAM flooding for cores of different permeability.

The results indicate that, whether in medium or relatively high permeability zones, the absolute incremental oil recovery of HPAM flooding improved that of water flooding by 12–17%. But, continuing HPAM flooding improved the absolute incremental oil recovery by 2.0–2.5% after HPAM flooding; switching to BD-HMHEC flooding improved the absolute incremental oil recovery by 7–8% after HPAM flooding. Therefore, it can be determined that the BD-HMHEC solution has a better oil displacement property than HPAM solution.

### The oil displacement mechanism of the BD-HMHEC solution

The oil recovery of BD-HMHEC flooding is much greater than those of HEC and HPAM flooding. Two possible oil displacement mechanisms to explain these results are given as follows.

#### The mechanism of enhanced viscosity

The BD-HMHEC solution exhibits good viscosification due to its hydrophobic association effect. It is generally considered that the apparent viscosity of polymer solution increases, the relative permeability of the water phase decreases and the water/oil mobility ratio reduces in porous media; thereby the oil displacement efficiency improves. A polymer’s ability to enhance oil recovery increases with increasing viscosity^37, 38^.

**Figure 9:**
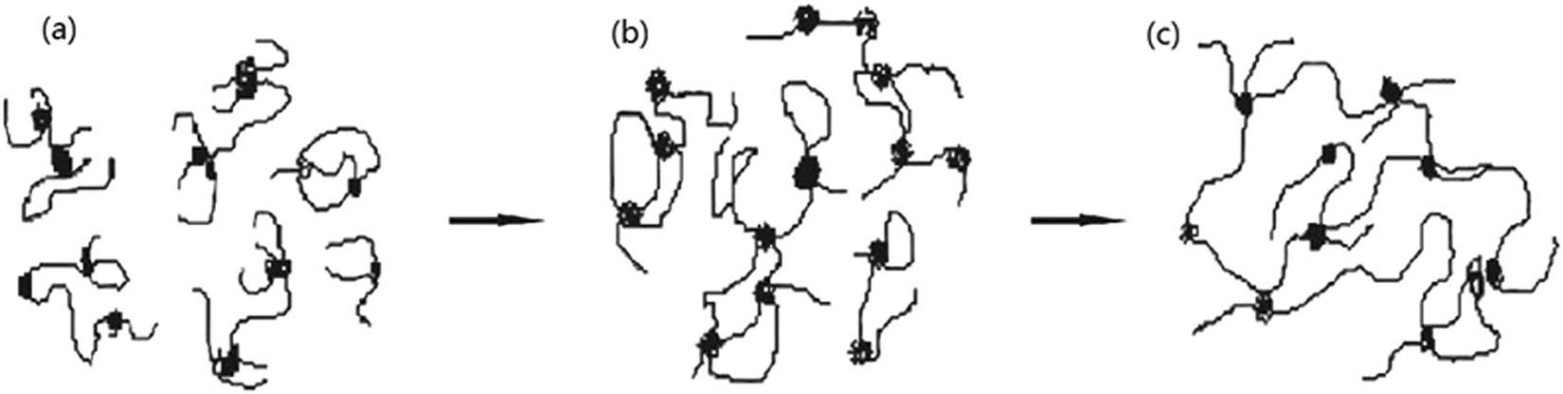
Schematic of the intermolecular association behavior of a BD-HMHEC solution with increasing concentration.

Figure 9 presents a schematic of the intermolecular association behavior of a BD-HMHEC solution with increasing concentration. The viscosification performance of the BD-HMHEC solution was poor at a relatively low concentration (i.e., for a concentration lower than *C*_p_^*^). The reason for this is that the macromolecular chains rely mainly on the intramolecular association of the hydrophobic groups, as shown in Fig. 9(a) and (b). When the concentration is greater than *C*_p_^*^, the macromolecular chains form a supermolecular structure (also denoted as a dynamic physical crosslinking network structure) with an intermolecular hydrophobic interaction base, as shown in Fig. 9(c). Supramolecular aggregates and hydrophobic regions are formed owing to association among the hydrophobic groups; thus, the solution viscosity increases significantly. However, HEC lacks these aggregates and structure regions. Therefore, BD-HMHEC solutions exhibit better viscosification and oil displacement performances than HEC solutions.

#### The mechanism of viscoelasticity

**Figure 10:**
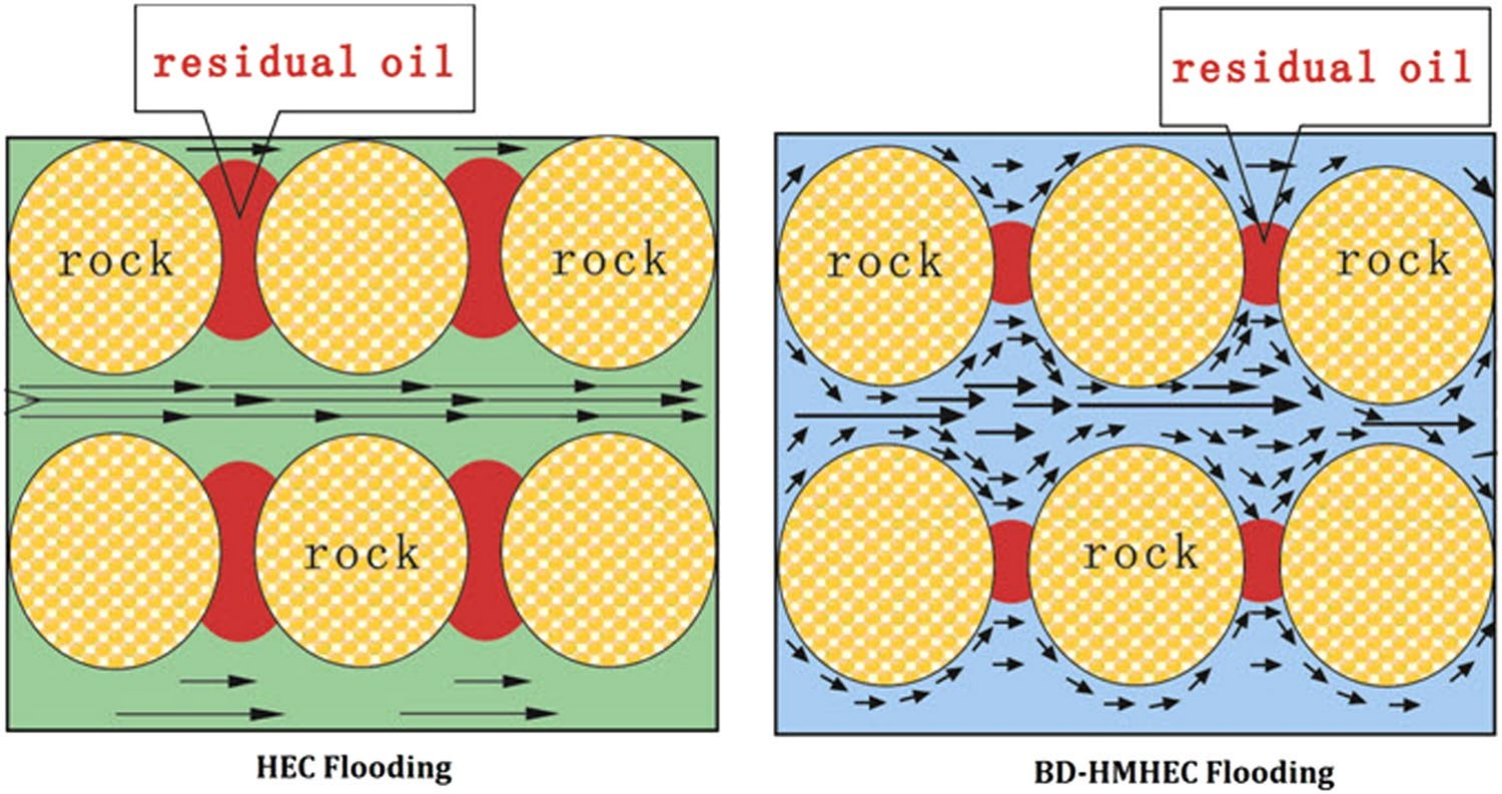
A schematic of the flooding process of oil droplets in water-wet cores.

**Figure 11:**
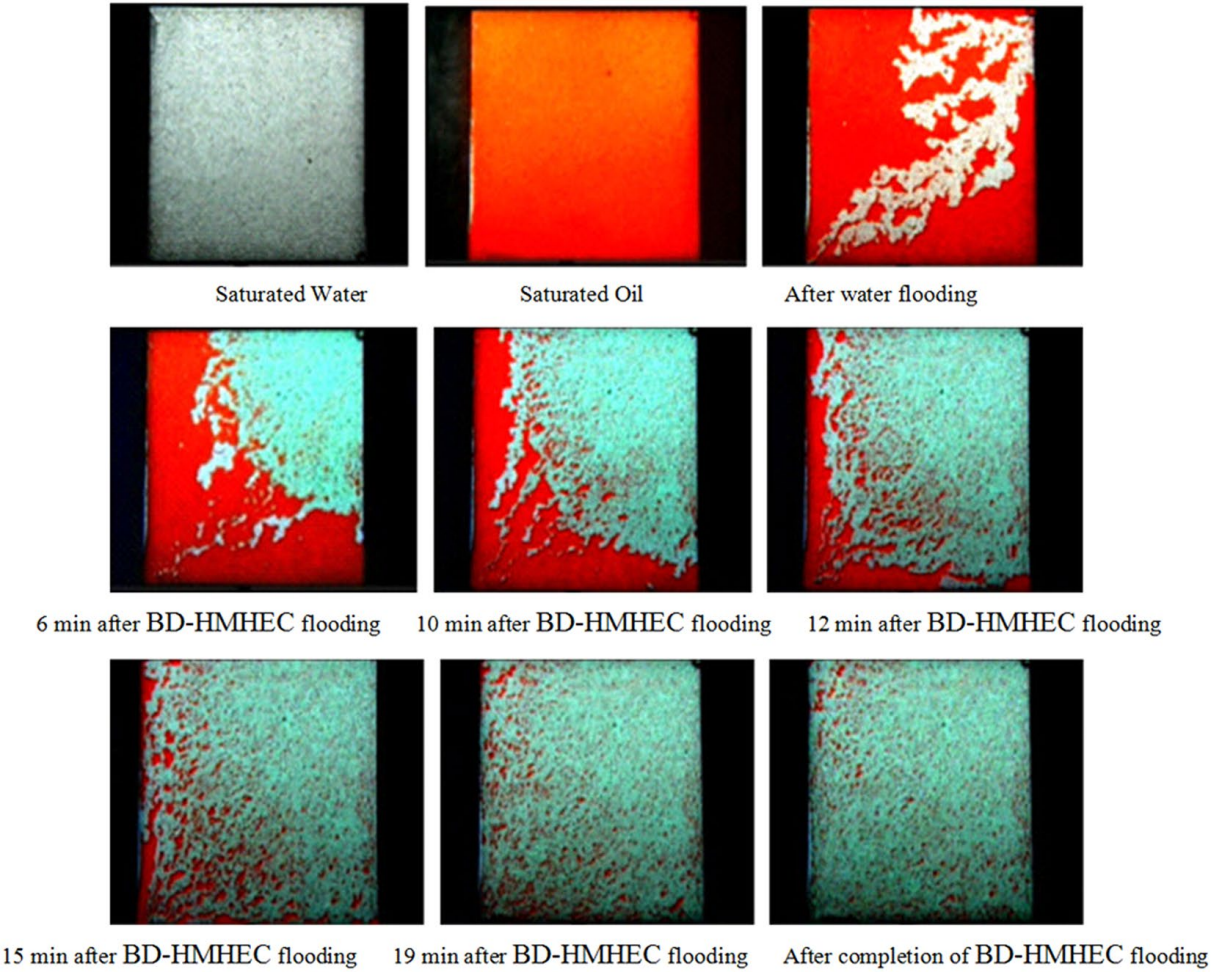
Visual evaluation of BD-HMHEC solution oil displacement.

The unique dynamic physical crosslinking network structure of a BD-HMHEC solution imparts a viscoelasticity to the solution, enabling it to “pull, drag” the residual oil in dead-end and pore throat, but the HEC solution lacks this characteristic^37, 38^. Figure 10 describes a schematic of the flooding process of oil droplets in water-wet cores. Figure 11 describes the visual evaluation of BD-HMHEC solution oil displacement (concentration: 4000 mg/L; temperature 25 °C). The viscoelasticity oil displacement mechanism of a BD-HMHEC solution is discussed as follows based on the incremental oil recovery determined according to the visual evaluation.

From Fig. 10, when the BD-HMHEC solution flows in a porous medium, pore throat, etc., the molecule space network structure is destroyed under the conditions of shear flow. However, when the flow path diameter or flow velocity changes, the space network structure is reconstituted, and the viscosity is restored due to its viscoelasticity. Meanwhile, as the partial flow resistance increases, the subsequent injected fluid is able to flow into narrow neck regions, and, at this point, the oil groups within the narrow zone form a process deformation along the flow direction with projecting parts that become detached from the main oil groups into the moveable oil, which draws off the residual oil. Thus, the BD-HMHEC solution exhibits EOR by improving reservoir displacement efficiency^11, 37–40^.

From Fig. 11, the experimental results indicate that the mean oil recovery of water flooding was about 28%, and that the total mean oil recovery was about 95% after BD-HMHEC flooding, resulting in about a 67% absolute incremental improvement over water flooding. The captured images show that BD-HMHEC improves the displacement efficiency because of its increased viscosity and viscoelasticity.

## Conclusion

1. The BD-HMHEC solution has good viscosification, thermal-resistance, salt-tolerance, shear resistance, and acid/alkali resistance.
2. The BD-HMHEC solution exhibits viscoelasticity when its concentration is greater than or equal to 4000 mg/L, and *G*′, *G*″, and the viscoelasticity all increase with increasing oscillating shear stress frequency.
3. Coreflood experimental results clearly indicated that the absolute incremental oil recovery of BD-HMHEC flooding was about 7–14% higher than that of HEC flooding under equivalent conditions. Moreover, BD-HMHEC flooding improved the incremental oil recovery by about 7–8% after HPAM flooding. These results demonstrate that BD-HMHEC flooding has much better oil displacement properties than those of HPAM and HEC flooding.
4. The visual experimental results indicated that the mean oil recovery of water flooding was about 28%, and the total mean oil recovery was about 95% after BD-HMHEC flooding, which achieved an improved absolute incremental oil recovery by about 67% relative to that of water flooding.
5. BD-HMHEC, as an oil displacement agent, can improve the oil displacement efficiency because of its viscosification and viscoelasticity, and has great potential in EOR processes.
